# Impact of clonal hematopoiesis of indeterminate potential on treatment outcomes in multiple myeloma patients undergoing CAR-T cell therapy

**DOI:** 10.1007/s12185-026-04249-4

**Published:** 2026-07-18

**Authors:** Sophie G. Carlson, Benjamin R. Puliafito, Kosuke Kawai, T. Niroshi Senaratne, Thomas D. Lee, Tasha Lin, John Timmerman, Sarah M. Larson

**Affiliations:** 1 Department of Medicine, Division of Hematology/Oncology, UCLA, Los Angeles, CA USA; 2 Department of Medical Oncology, Massachusetts General Hospital, Boston, MA USA; 3 Department of Medicine Statistics Core, UCLA, Los Angeles, CA USA; 4 Department of Pathology and Laboratory Medicine, UCLA, Los Angeles, CA USA; 5 Department of Pathology & Laboratory Medicine, School of Medicine, University of California, Irvine, CA USA

**Keywords:** CHIP, Multiple myeloma, CAR-T

## Abstract

Clonal hematopoiesis of indeterminate potential (CHIP) is the presence of hematologic malignancy-associated somatic mutations in patients without hematologic malignancies. In multiple myeloma (MM), the implications of CHIP on chimeric antigen receptor (CAR)-T cell outcomes and toxicities have yet to be fully explored. In a retrospective study at UCLA, we explored the association between CHIP and clinical outcomes of 68 adult patients with MM (31% with CHIP mutations) who were undergoing CAR-T cell therapy from 6/1/2021 to 12/31/2024. The overall (95% CHIP vs. 93% no CHIP; *p* = 0.99) and complete response rates (81% CHIP vs. 67% no CHIP; *p* = 0.15) did not differ by CHIP status. Similarly, CHIP mutations did not impact progression-free survival (HR 0.83; 95% CI 0.38–1.83; *p* = 0.65) or overall survival (HR 0.76; 95% CI 0.24–2.45; *p* = 0.65). Patients with CHIP mutations were more likely to develop CRS (100% vs. 70%; *p* = 0.007); however, there was no difference in the incidence of ICANS (33% CHIP vs. 17% no CHIP, *p* = 0.20). Our results show that although CHIP did not negatively influence response rate, complete response rate, survival, or progression-free survival in MM patients after CAR-T cell therapy, it did confer a higher risk of inflammatory response with increased frequency and grade of CRS.

**Supplementary Information:**

The online version contains supplementary material available at https://doi.org/10.1007/s12185-026-04249-4.

## Introduction

Clonal hematopoiesis of indeterminate potential (CHIP) is the presence of hematologic malignancy-associated somatic mutations that drive hematopoietic stem cell clonal expansion and are seen in patients without hematologic malignancy, dysplasia, or cytopenias [1]. CHIP mutations are most commonly acquired during human aging and are known to be associated with an increased risk of progression to a hematologic malignancy at a rate of 0.5 to 1% per year, in particular myelodysplastic syndrome (MDS) and acute myeloid leukemia (AML), and with the risk generally proportional to the size of the somatic clone [2]. Further, CHIP is also correlated with inflammatory cytokines and with pro-inflammatory states that have been linked to increased risk of diseases such as coronary artery disease, venous thromboembolic disease, stroke, and heart failure [3–6].

The clinical significance of CHIP in patients with multiple myeloma is currently being explored. In a prior study of 76 patients with newly diagnosed multiple myeloma, 46% of patients had CHIP, with the most frequently mutated gene being DNMT3A, followed by TET2. This study found that more aggressive disease features were observed among patients with CHIP, including larger proportion of high-risk stages [7]. Another such study by Gagnon et al. detected CHIP in 23% of patients with multiple myeloma. This study also demonstrated that the presence of CHIP negatively impacted overall survival in black patients with multiple myeloma (HR = 4.57, 95% CI 1.48–14.1; *p* = 0.008) as well as worse progression-free survival [8]. In a large cohort of 600 patients with multiple myeloma treated with an autologous stem cell transplant (ASCT), 21.6% of patients were found to have CHIP, and the presence of CHIP at the time of ASCT conferred worse clinical outcomes: The median overall survival (OS) of patients with CHIP was 5.3 years, significantly lower than in those without CHIP (7.5 years, HR: 1.34, *p* = 0.02) and the median progression-free survival (PFS) was 2.2 years in patients with CHIP vs. 2.6 years without CHIP (HR: 1.45, *p* < 0.001) [9]. This influence of CHIP on disease progression and response after transplant, highlights the importance to further understand the relationship between CHIP and other myeloma directed therapy [10].

Chimeric antigen receptor (CAR)-T cell therapy revolutionized treatment options for patients with multiple myeloma with impressive overall response rates (ORRs) and progression-free survival (PFS). In the first CARTITUDE-1 trial of heavily pre-treated patients with multiple myeloma, the overall response rate was 97% and with a median follow-up of 61.3 months, the median overall survival was 60.7 months [11, 12]. The CARTITUDE-4 trial compared cilta-cel to standard of care in lenalidomide-refractory multiple myeloma, and the overall response rate was 84.6% with a progression-free survival at 12 months of 75.9%, as well as 30-month overall survival of 76.5% in cilta-cel arm compared to 63.8% in the standard of care arm [13, 14]. CAR-T cell therapy has become ubiquitous in the multiple myeloma treatment paradigm, and yet little is understood about the relationship between CHIP and CAR-T cell therapy outcomes in patients with multiple myeloma. Because CHIP is associated with inflammation, it is worth exploring the interaction between CHIP and CAR-T outcomes, which has been done with CD19-directed CAR-T cells in patients with relapsed/refractory diffuse large B cell lymphoma. In one study by Miller et al., among 144 lymphoma patients who received CD19-directed CAR-T cell therapy, CHIP was associated with increased rates of CRS grade ≥ 2 without a difference in progression-free or overall survival [15]. In contrast, a smaller study by Teipel et al., 32 patients with lymphoma received CD19-directed CAR-T cell therapy and did not demonstrate any differences in CRS/ICANS, hematopoietic recovery, or survival/response outcomes [16].

With the rise in the use of CAR-T cell therapy in multiple myeloma, and the prevalence of CHIP among patients, it is becoming increasingly important to explore their relationship. In this study, we explored the impact of CHIP mutations on clinical outcomes in patients with multiple myeloma undergoing CAR-T cell therapy at a single institution.

## Methods

### Study design

This study was approved by UCLA Institutional Review Board as a retrospective, single center study at UCLA Medical Center. A total of 68 adult patients with multiple myeloma who received commercial, standard of care CAR-T cell therapy between 6/1/2021 and 12/31/2024 were included in the study. Subjects were required to have available next-generation sequencing (NGS) data from a bone marrow sample within 1 year prior to CAR-T cell infusion. Patients were excluded if they had a prior diagnosis of myelodysplastic syndrome or acute myeloid leukemia. Treatment responses were assessed per the International Myeloma Working Group criteria. CRS and ICANS were graded according to the American Society for Transplantation and Cellular Therapy guidelines. The study design complied with the Declaration of Helsinki and International Conference on Harmonization Guidelines for Good Clinical Practice. All patients underwent informed consent per institutional guidelines and in accordance with the Declaration of Helsinki.

### Genomic studies

CHIP mutations were defined as somatic mutations with VAF ≥ 2% and < 40%, and we excluded KRAS, NRAS, EZH2, and TP53 mutations, as we did not do additional sequencing to confirm that it was not related to underlying disease. CHIP testing was performed on patient bone marrow samples within one year prior to CAR-T cell therapy and post CAR-T cell therapy. DNA was isolated from the specimen by standard methods and subjected to targeted amplification of multiple coding regions of 54 genes using the Illumina TruSight Myeloid Sequencing Panel. The genes are: *ABL1*, *ASXL1*, *ATRX*, *BCOR*, *BCORL1*, *BRAF*, *CALR*, *CBL*, *CBLB*, *CBLC*, *CDKN2A*, *CEBPA*, *CSF3R*, *CUX1*, *DNMT3A*, *ETV6*, *EZH2*, *FBXW7*, *FLT3*, *GATA1*, *GATA2*, *GNAS*, *HRAS*, *IDH1*, *IDH2*, *IKZF1*, *JAK2*, *JAK3*, *KDM6A*, *KIT*, KMT2A, *KRAS*, *MPL*, *MYD88*, *NOTCH1*, *NPM1*, *NRAS*, *PDGFRA*, *PHF6*, *PTEN*, *PTPN11*, *RAD21*, *RUNX1*, *SETBP1*, *SF3B1*, *SMC1A*, *SMC3*, *SRSF2*, *STAG2*, *TET2*, *TP53*, *U2AF1*, *WT1*, and *ZRSR2*. Following next-generation sequencing on the Illumina MiSeq, the raw sequencing data were processed with the on-board Real-Time Analysis (RTA) pipeline to identify and annotate sequence alterations. This assay can reliably and reproducibly detect approximately 5% of an alternate (mutant) single nucleotide variant allele, or 10% of an insertion/deletion allele, in a background of reference (wild-type) alleles, although lower alternate allele fractions can occasionally be detected. This method is limited to the detection of certain categories of mutations, including single nucleotide variants and small insertions/deletions (typically defined as ≤ 20 nucleotides), within the regions of the genome targeted by this assay. The assay is designed to test for somatic alterations only, however, occasional presumed germline alterations may be detected. These alterations need to be confirmed by testing germline-derived specimens. VAF > 40% was presumed to be germline mutation and was not considered CHIP.

### Statistical analysis

Fisher’s exact or Chi-squared test was conducted to compare the response rate between patients with CHIP vs. those without CHIP. Kaplan–Meier survival plots were constructed to examine the progression-free survival (PFS) and the overall survival (OS). We used the Cox proportional hazards model to examine the PFS by CHIP mutation. Multivariable Cox proportional hazards models were built to adjust for extra-medullary disease and high-risk cytogenetics. Hazard Ratio (HR) and 95% confidence interval (CI) were estimated. All analyses were performed using SAS version 9.4 (SAS Institute, Cary, NC) and Stata version 19 (StataCorp, College Station, TX).

## Results

### Patient characteristics

**Fig. 1:**
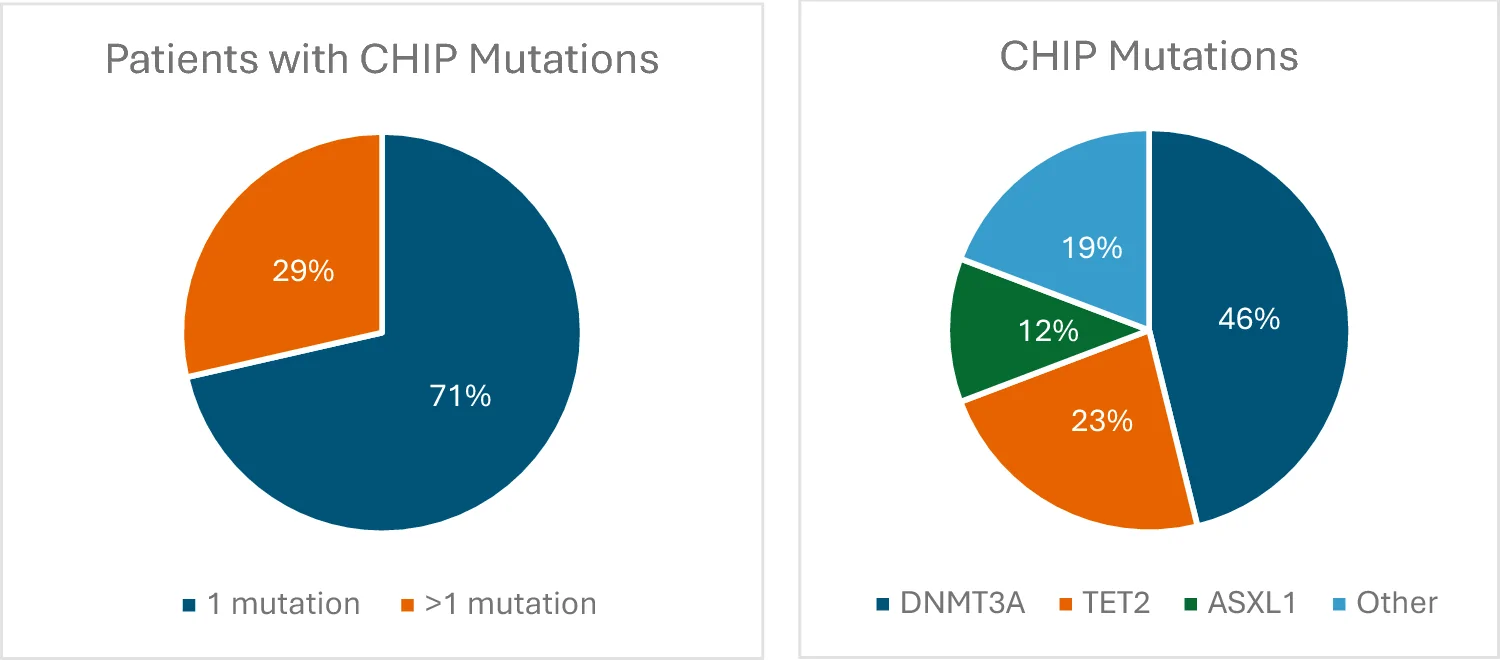
Landscape of CHIP mutations among patients with multiple myeloma who received CAR-T cell therapy

A total of 71 adult patients with multiple myeloma were identified who received CAR-T cell therapy. Three patients were excluded from the study who either lacked NGS data prior to treatment or received CAR-T cell therapy as a part of a clinical trial. Of the 68 eligible patients, a CHIP mutation was present in 21 patients. Baseline characteristics are summarized in Table 1. The mean age at the time of CAR-T cell therapy was 63.9 years (SD 9.0; range 40 to 78). The mean age of the patients did not differ by CHIP mutation status (mean 66 CHIP vs. 62.9 no CHIP; *p* = 0.18). Forty-seven percent of the overall cohort was female. The median number of prior lines of therapy among both groups was 4 (ranges 2 to 11), with 66% and 81% having received an autologous stem cell transplant prior to CAR-T cell therapy among patients with no CHIP and CHIP, respectively. More patients without CHIP mutations received chemotherapy (65%) compared to those with CHIP mutations (48%), and the number of patients who received lenalidomide, bortezomib, and anti-CD38 monoclonal antibody was similar between groups. The presence of high-risk cytogenetics by IMWG recommendations was similar between groups (45% with CHIP vs. 50% no CHIP) (Table 1) [17]. Regarding the CHIP mutation cohort, a single CHIP mutation was detected in 15/21 patients, while 6 patients had two or more mutations in different genes (Fig. 1). The most common CHIP mutation was in the gene DNMT3A followed by TET2 and ASXL1 (12, 6, and 3 patients, respectively) (Table 2).

**Table 1:** Baseline characteristics of patients with and without CHIP

	All patients (*n* = 68)	CHIP (*n* = 21)	No CHIP (*n* = 47)
Age, mean (SD)	63.9 (9.0)	66.1 (7.3)	62.9 (9.5)
Female, n (%)	32 (47%)	9 (43%)	23 (49%)
High-risk cytogenetics, n (%)	32 (48%)	9 (45%)	23 (50%)
CNS involvement, n (%)	3 (4%)	0 (0%)	3 (6%)
Extramedullary disease, n (%)	25 (37%)	9 (42%)	16 (24%)
Time since diagnosis in yrs, median (range)	4.74 (0.71–18.9)	4.74 (0.71–12.7)	4.61 (0.74–18.9)
Number of prior lines of therapy, median (IQR)	4 (3–6)	4 (3–5)	4 (3–6)
Previous therapies, n (%)			
Bortezomib	67 (99%)	21 (100%)	46 (98%)
Carfilzomib	58 (85%)	20 (95%)	38 (81%)
Ixazomib	15 (22%)	7 (33%)	8 (17%)
Lenalidomide	68 (100%)	21 (100%)	47 (100%)
Pomalidomide	52 (76%)	18 (86%)	34 (72%)
Anti-CD38 monoclonal Ab	66 (97%)	20 (95%)	46 (98%)
Bi-specific Ab	3 (4%)	0 (0%)	3 (6%)
Chemotherapy	41 (60%)	10 (48%)	31 (65%)
Autologous transplant prior to CAR-T, n (%)	48 (71%)	17 (81%)	31 (66%)
Bridging therapy after leukapheresis, n (%)	44 (66%)	14 (70%)	30 (64%)
Lymphodepletion with Flu/Cy	67 (99%)	20 (95%)	47 (100%)
CAR-T cell product, n (%)			
Idecabtagene vicleucel	22 (32%)	7 (33%)	15 (32%)
Ciltacabtagene autoleucel	46 (68%)	14 (67%)	32 (68%)

Following variables with missing values: cytogenetics (*n* = 2) and bridging therapy (*n* = 1)

High-risk cytogenetics by IMWG 2025 recommendations

**Table 2:** CHIP mutations identified among patients with multiple myeloma who received CAR-T cell therapy

Gene	Number of patients with mutation (%)*
DNMT3A	12 (57)
TET2	6 (28)
ASXL1	3 (14)
SETBP1	1 (5)
RAD21	1 (5)
CBL	1 (5)
SMC3	1 (5)
SF3B1	1 (5)

^*^Among patients with CHIP (*n* = 21)

### Outcomes

**Fig. 2:**
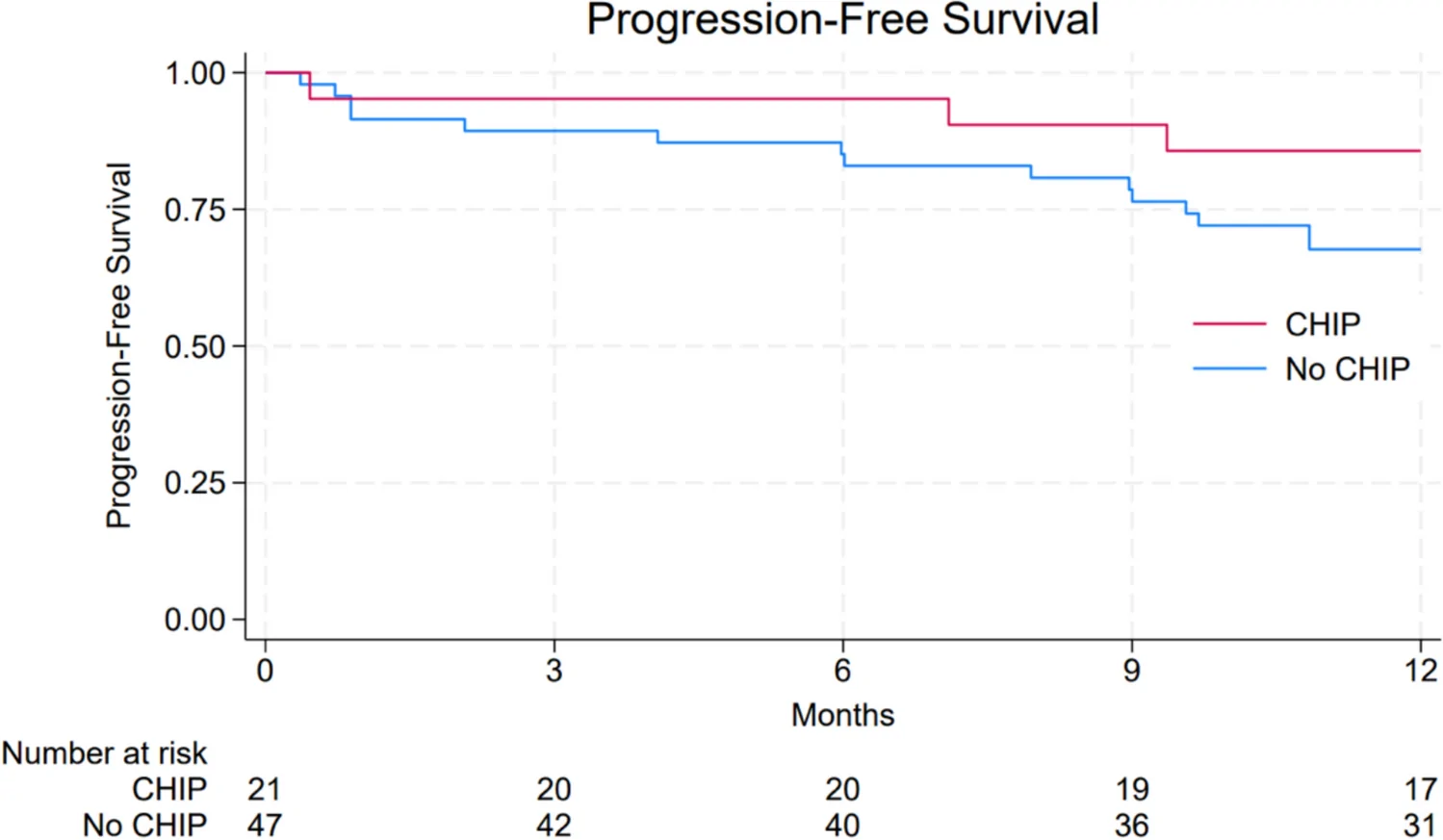
Progression-free survival of patients with or without CHIP

**Fig. 3:**
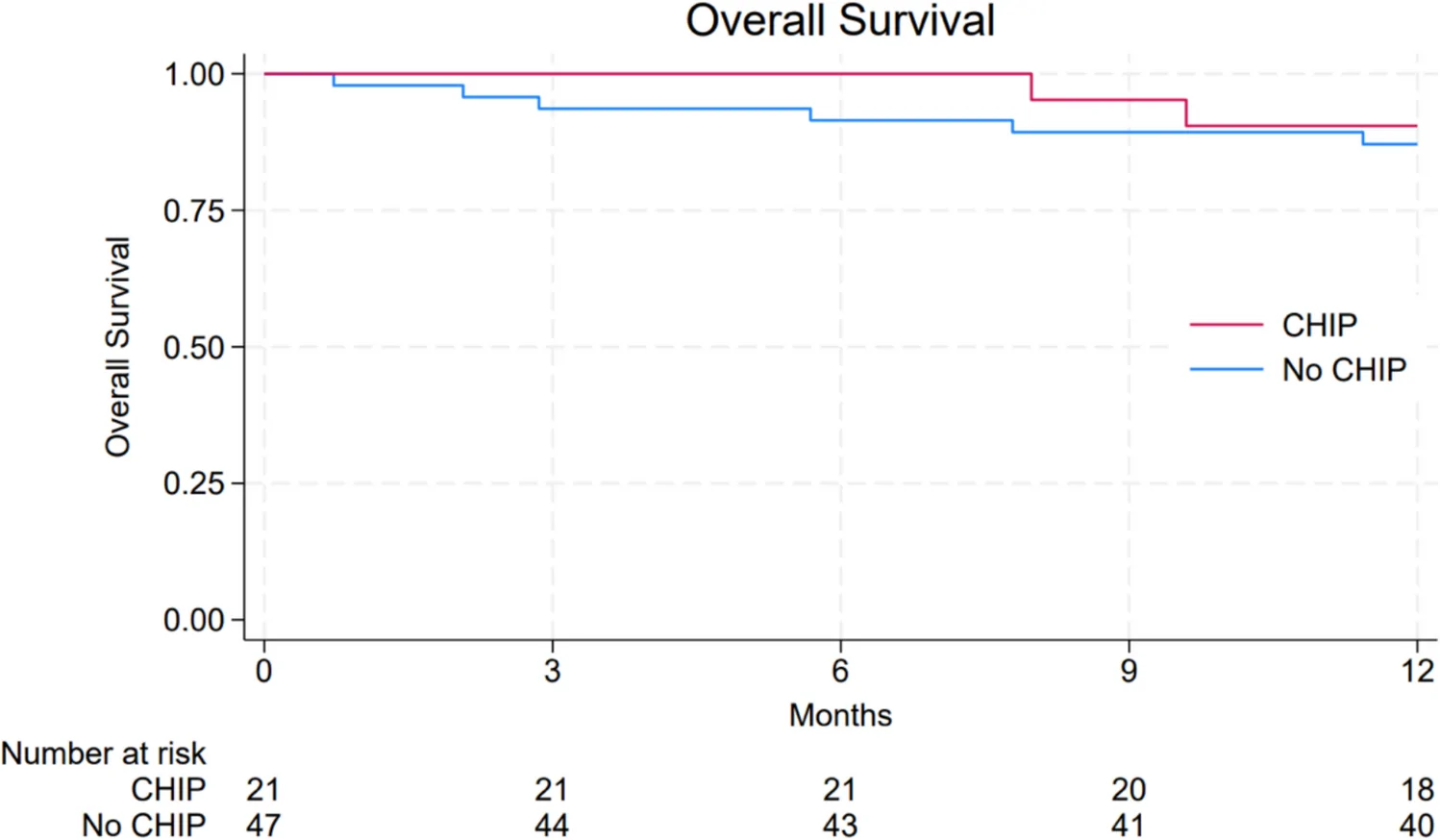
Overall survival of patients with or without CHIP

The overall response rate in patients undergoing CAR-T cell therapy did not differ by the presence of CHIP mutations (95% in CHIP vs. 93% in no CHIP; *p* = 0.99). The complete response rate also did not differ by CHIP status (81% in CHIP vs. 67% in no CHIP; *p* = 0.15) (Table 3). With a mean follow-up time was 20 months (SD 10), the presence of CHIP mutations was also not associated with PFS (HR 0.83; 95% CI 0.38–1.83; *p* = 0.65). The 1-year PFS was 85% (95% CI 61–95%) in patients with CHIP mutations, whereas 1-year PFS 68% (95% CI 52–79%) in patients without CHIP mutations. The presence of CHIP also did not affect the overall survival (HR 0.76; 95% CI 0.24–2.45; *p* = 0.65). The 1-year OS was 90% (95% CI 66–97%) in patients with CHIP mutations vs. 87% (95% CI 74–94%) in patients without CHIP mutations (Fig 2 and 3). In a multivariate analysis that included extramedullary disease and high-risk cytogenetics, the CHIP mutations were not associated with PFS (adjusted HR 0.84; 95% CI 0.38–1.86; *p* = 0.67) or OS (adjusted HR 0.70; 95% CI 0.21–2.28; p = 0.55) (Supplemental Table 2). The association between CHIP and clinical outcomes were also similar between CAR-T cell products (Supplemental Table 1).

**Table 3:** Clinical outcomes by CHIP mutations in patients with multiple myeloma who received CAR-T cell therapy

	CHIP (*n* = 21)	No CHIP (*n* = 47)	*P* value
*Best response*			0.63
CR	0 (0%)	1 (2%)	
sCR	18 (85%)	30 (65%)	
PR	0 (0%)	2 (4%)	
VGPR	2 (10%)	10 (22%)	
SD	0 (0%)	0 (0%)	
PD	1 (5%)	3 (7%)	
Response	20 (95%)	43 (93%)	0.99
Complete response (CR/sCR)	17 (81%)	31 (67%)	0.15
Inflammatory markers, mean (SD)			
Ferritin D0	420.5 (573.5)	724.4 (1190.7)	0.61
Max Ferritin (to D28)	1798.4 (2002.1)	4122.4 (12,862.5)	0.41
Change in Ferritin (D0-D28)	1378.0 (1760.3)	3398.0 (12,450.6)	0.39
CRP D0	0.7 (1.8)	1.3 (2.8)	0.49
Max CRP (to D28)	5.5 (3.5)	7.0 (6.0)	0.63
Change in CRP (D0-D28)	4.8 (3.2)	5.7 (5.2)	0.78
CRS occurrence			
All grade	21 (100%)	33 (70%)	0.003
Maximum grade 1/2	20 (95%)	32 (68%)	
Maximum grade 3 +	1 (5%)	1 (3%)	
ICANS occurrence			
All grade	7 (33%)	8 (17%)	0.20
Maximum grade 1/2	7 (33%)	6 (13%)	
Maximum grade 3 +	0 (0%)	1 (2%)	
Received tocilizumab for CRS or neurotoxicity treatment	19 (90%)	28 (60%)	0.01
Non-ICANS neurotoxicity	4 (19%)	6 (13%)	0.49
Cranial nerve palsy	4 (19%)	3 (6%)	
Other	0 (0%)	3 (6%)	
Parkinsonian symptoms	1 (0.5%)	1 (0.02%)	
Secondary myeloid malignancy	2 (0.1%)	0 (0%)	

One patient with unknown response, one with unknown ICANS grade

Patients with CHIP mutations were more likely to develop CRS than those without CHIP mutations (100% vs. 70%; *p* = 0.007). Specifically, those with CHIP mutations had more max grade 1/2 CRS (95% CHIP vs. 68% no CHIP), while the amount of max grade 3 + CRS was similar (5% CHIP vs. 3% no CHIP) between groups. The incidence of ICANS was 33% in patients with CHIP vs. 17% in patients without CHIP (*p* = 0.20). No patients with CHIP mutations had ICANS grade 3 + , while one patient in the no CHIP mutation group had ICANS grade 3 + . There was no difference in non-ICANS neurotoxicity (19% vs. 13%, *p* = 0.49). All four patients with CHIP mutation who developed non-ICANS neurotoxicity had a cranial nerve seven palsy, while three out of six of the patients with no CHIP mutation developed a cranial nerve seven palsy. There was also one patient in each group that developed Parkinsonian symptoms. Patients with CHIP mutations were more likely to receive tocilizumab for CRS or neurotoxicity (90% vs. 60%; *p* = 0.01) (Table 3). The mean change in absolute lymphocyte count between days -5 and day 14 was greater in patients with CHIP (4.29 with CHIP vs. 1.83 without CHIP, *p* = 0.0025) (Table 4) (Figs. 2 and 3).

**Table 4:** Cytopenia data post CAR-T cell therapy

	CHIP (*n* = 21)	No CHIP (*n* = 47)	*P* value
Mean absolute lymphocyte change (day -5 to day 14)	4.29	1.83	0.0025
*Max grade anemia (D28-90)*			0.82
Grade 0	1 (5.9%)	3 (8.6%)	
Grade 1	7 (41.2%)	13 (37.1%)	
Grade 2	5 (29.4%)	8 (22.9%	
Grade 3	4 (23.5%)	11 (31.4%)	
Grade 4	0	0	
*Max grade thrombocytopenia (D28-90)*			0.45
Grade 0	1 (5.9%)	7 (20.0%)	
Grade 1	6 (35.3%)	8 (22.9%)	
Grade 2	1 (5.9%)	5 (14.3%)	
Grade 3	4 (23.5%)	7 (20.0%)	
Grade 4	5 (29.4%)	8 (22.9%)	
*Max grade neutropenia (D28-90)*			0.44
Grade 0	3 (17.7%)	9 (26.5%)	
Grade 1	0 (0%)	1 (2.9%)	
Grade 2	2 (11.8%)	5 (14.7%)	
Grade 3	6 (35.3%)	7 (20.6%)	
Grade 4	6 (35.3%)	12 (35.3%)	
*Max grade anemia (D91-120)*			0.32
Grade 0	5 (29.4%)	7 (21.2%)	
Grade 1	8 (47.1%)	14 (42.4%)	
Grade 2	3 (17.7%)	8 (24.2%)	
Grade 3	1 (5.9%)	4 (12.1%)	
Grade 4	0 (0%)	0 (0%)	
*Max grade thrombocytopenia (D91-120)*			0.28
Grade 0	6 (35.3%)	8 (24.2%)	
Grade 1	5 (29.4%)	12 (36.4%)	
Grade 2	4 (23.5%)	4 (12.1%)	
Grade 3	1 (5.9%)	3 (9.1%)	
Grade 4	1 (5.9%)	6 (18.2%)	
*Max grade neutropenia (D91-120)*			0.86
Grade 0	10 (58.8%)	16 (50%)	
Grade 1	0 (0%)	2 (6.3%)	
Grade 2	2 (11.8%)	6 (18.8%)	
Grade 3	4 (23.5%)	6 (18.8%)	
Grade 4	1 (5.9%)	2 (6.3%)	
Use of GCSF between D90 and D120	5 (20%)	2 (5%)	0.033
Use of TPO mimetic between D90 and D120	0 (0%)	5 (11.9%)	0.16
Use of epo between D90 and D120	0 (0%)	0 (0%)	
pRBC transfusion between D90 and D120	1 (5%)	5 (12.5%)	0.65
Use of GCSF between D120 and D365	6 (32.6%)	5 (11.9%)	0.08
Use of TPO mimetic between D120 and D365	2 (10.5%)	7 (16.7%)	0.70
Use of epo between D120 and D365	0 (0%)	0 (0%)	
pRBC transfusion between D120 and D365	1 (5.6%)	7 (18%)	0.41

Patients not included in D90-365 if started new therapy or deceased

There was no difference in early (day 28–90) or late (day 91–120) anemia, thrombocytopenia, or neutropenia based on CHIP status. However, between day 90 and day 120, G-CSF was used more among patients with CHIP mutations (20% in CHIP vs. 5% without CHIP, *p* = 0.03), as well as between days 120–365 (32.6% in CHIP vs. 11.9% without CHIP, *p* = 0.08). There was overall very limited use of tpo mimetics or epo agonist, and there was no difference in the early or late use of a TPO mimetic or epo use between groups (Table 4). Although the long-term follow-up data are limited, two patients with CHIP mutations developed a secondary myeloid malignancy. One patient developed high-risk myelodysplastic syndrome (MDS) by IPSS-M. Prior to CAR-T cell therapy this patient had DNMT3A mutation in exon 19 and exon 22 with a VAF of 2% and 4%. At the time of the MDS diagnosis, they developed new TP53 mutations with VAF of 43% and 45% with loss of the prior DNMT3A mutation. The other patient, who initially had a DNMT3A mutation with VAF of 6% as well as a TP53 mutation with VAF of 18%, also developed MDS (Table 3).

## Discussion

Thus far, the potential impact of CHIP on outcomes among patients with multiple myeloma receiving CAR-T cell therapy has not been well-studied. As more patients receive CAR-T cell therapy, either for relapsed/refractory disease or potentially in the frontline setting driven by trials such as CARTITUDE-6 (NCT05257083), it will become even more important to understand the impact of CHIP on post-CAR-T cell outcomes. With CAR-T demonstrating an overall survival benefit in CARTITUDE-4, with 30-month overall survival of 76.5% in cilta-cel arm compared to 63.8% in the standard of care arm, as well as a 70% reduction in risk of progression or death in patients who received cilta-cel and significantly increased overall MRD negativity rates, patients are living longer post CAR-T cell therapy and it will be especially critical to understand the longer-term impacts of CHIP that affect quality of life such as secondary malignancies and non-ICANS neurotoxicity [14, 18, 19].

In this study, 30.9% of patients had a CHIP mutation, which is similar to previously published data reporting a range from 20 to 46%. Also consistent with prior studies, the most frequently mutated gene was DNMT3A, followed by TET2 [7, 8]. There were no significant differences in baseline characteristics between patients with and without CHIP mutations, including number of prior lines of therapy and autologous stem cell transplant. Overall, in this single institution study, we demonstrated that CHIP mutations are not associated with response rates (95% in CHIP vs. 93% in no CHIP; *p* = 1.0), overall survival (HR 0.76; 95% CI 0.24–2.45; *p* = 0.65), or progression-free survival (HR 0.83; 95% CI 0.38–1.83; *p* = 0.65). This is reassuring as it highlights the ongoing efficacy of CAR-T cell therapy despite the presence of CHIP mutations. This is supported by a recent study by Miller et al.: although only 10 multiple myeloma patients were included, they demonstrated that CHIP was not associated with a difference in progression-free or overall survival [15]. This is also consistent with what was seen in a larger study by Gustine et al. that included 104 patients, which also demonstrated no difference in responses, progression-free survival, or overall survival between patients with and without CHIP [20]. Interestingly, this differs from previously published data among patients with lymphoma and multiple myeloma undergoing autologous transplant, where CHIP mutations conferred worse outcomes [9, 21].Thus, as CAR-T is explored as possible first-line therapy in lieu of autologous transplant, outcomes data from this study suggests CAR-T cell therapy as a possible preferred treatment strategy for those patients with CHIP mutations.

Unlike response data which showed no differences among those with and without CHIP, there was a significant increase in the amount of CRS in those patients with CHIP mutations (100% vs. 70%, *p* = 0.003). Regardless of CHIP status, the majority of CRS was grade 1/2 in both groups (95% CHIP vs. 97% no CHIP). This increase in CRS was also reflected in the significantly increased frequency of tocilizumab given in the CHIP cohort (90% vs. 60%, *p* = 0.01). From a mechanistic and scientific standpoint, the observed association between increased CRS in patients with CHIP undergoing CAR-T cell therapy is biologically plausible, as CHIP is well established to promote a pro-inflammatory state. In humans, clonal hematopoiesis has been associated with elevated levels of inflammatory cytokines including IL-6, IL-8, and TNFα, with gene-specific associations—most notably *TET2* mutations correlating with increased IL-6 and DNMT3A mutations with elevated TNF*α* [22]. Indeed, in a pre-clinical study exploring how inflammatory signals influence hematopoietic stem and progenitor cells, researchers demonstrated that mice with TET2 mutations showed increased expression of IL-6 [23]. CAR-T cell therapy has a robust inflammatory response associated with the presence of high levels of pro-inflammatory cytokines, many of which overlap with those seen with CHIP [24, 25]. Despite this, in a recently published study by Gustine et al. exploring the impact of CHIP on CAR-T cell outcomes in patients with multiple myeloma, they did not see a significant difference in the incidence of CRS based on CHIP status. The reason for the difference in CRS outcomes between these two studies is unclear; however, in the Gustine et al. study, the majority of patients received either ide-cel or investigational drug compared to this study where the majority of patients received cilta-cel [20].

Although there was a trend toward more ICANS among those with CHIP mutations, it was not statistically significant (33% vs. 17%, *p* = 0.2). There was also no difference in non-ICANS neurotoxicity (19% vs. 13%, *p* = 0.49), with the most common non-ICANS neurotoxicity being cranial nerve palsy (7/10 patients). There were two cases of motor and neurocognitive toxicity split evenly between the two cohorts of (CHIP 1/21 vs. No CHIP 1/47). Interestingly, a more recent observation among patients receiving CAR-T cell therapy is a correlation between increased absolute lymphocyte count and neurotoxicity. In a retrospective study using the IMWG registry, investigators noted that amongst the 37 patients that had cranial nerve palsies, the median peak ALC was 4.1 × 10^9^/L and among the 16 cases of Parkinson’s disease the peak ALC was 9.5 × 10^9^/L [26]. This is consistent with another study from Colorado Blood Cancer Institute which showed that patients with an unrestrained ALC > 7 × 10^9^/L had higher rates of atypical neurotoxicity events [27]. However, in this study despite the mean change in absolute lymphocyte count being greater in patients with CHIP (4.29 with CHIP vs. 1.84 without CHIP, *p* = 0.0025), there was not a higher incidence of non-ICANS neurotoxicity among CHIP patients. It is possible this discrepancy is due to the method of data collection, specifically in that we did not evaluate the difference using a peak ALC threshold. Finally, despite increased use of G-CSF between day 90 and 120 among the CHIP group, there was no difference in cytopenias based on CHIP status.

This retrospective study is not without limitations, particularly in the identification and definition of CHIP mutations. For the purposes of this study, we excluded certain mutations such as TP53 among others, as well as VAF less than 2% and greater than 40%. The exclusion of mutations with a VAF greater than 40% was based on the general assumption that such mutations are likely germline rather than somatic. While the Illumina TruSight Myeloid Panel is a valuable tool for detecting mutations, it may miss mutations outside of the panel’s targeted regions, as well as other clinically relevant mutations that are not covered. Additionally, the technology is susceptible to both false positives and false negatives, especially for low allele frequency mutations common in CHIP. Furthermore, this retrospective study is limited to a single center and thus has potential bias in patient selection that may limit the generalizability of this study.

Overall, similar to recently published data exploring CAR-T cell outcomes in patients with multiple myeloma, this study demonstrates that CHIP is not associated with response rate, progression-free survival, or overall survival. There are increased rates of CRS, which is not unexpected given the known association of CHIP with inflammation, but the majority of CRS remains grade 1/2. This study demonstrates that CAR-T cell therapy exhibits a favorable safety profile and remains efficacious irrespective of CHIP status, an important finding as its use expands into earlier treatment lines and broader clinical use.

## Supplementary Information

Below is the link to the electronic supplementary material.

Supplementary file 1 (DOCX 18 kb)

## Data Availability

The data generated in this study are available within this publication or from the corresponding author upon reasonable request.
