# Supplementary material for: Impact of clonal hematopoiesis of indeterminate potential on treatment outcomes in multiple myeloma patients undergoing CAR-T cell therapy
Source: Int J Hematol. 2026 Jul 18;124(5):670–9. doi: 10.1007/s12185-026-04249-4 (PMC13719782; doi:10.1007/s12185-026-04249-4)
Supplement: Supplementary file 1 [file 12185_2026_4249_MOESM1_ESM.docx]

**Supplemental Tables and Figures**

**Supplemental Table 1**. Clinical outcomes by CHIP mutations and CAR-T cell product in patients with multiple myeloma.

|  | **Ciltacabtagene autoleucel** | | **Idecabtagene vicleucel** | |
| --- | --- | --- | --- | --- |
|  | **CHIP (n=14)** | **No CHIP (n=32)** | **CHIP (n=7)** | **No CHIP (n=15)** |
| Response | 14 (100%) | 32 (100%) | 6 (86%) | 11 (79%) |
| Complete response (CR/sCR) | 14 (100%) | 24 (75%) | 4 (57%) | 7 (50%) |
| CRS occurrence | 14 (100%) | 24 (75%) | 7 (100%) | 9 (60%) |
| ICANS occurrence | 4 (29%) | 3 (9%) | 3 (43%) | 5 (33%) |

* One patient with unknown response (ide-cel, no CHIP)

**Supplemental Table 2**. Multivariable Cox proportional hazards models were used to adjust for extra-medullary disease and high-risk cytogenetics.

| **Outcome** | **Covariate** | **HR** | **95% CI** | **p-value** |
| --- | --- | --- | --- | --- |
| Progression Free Survival | CHIP vs. No CHIP | 0.84 | 0.38-1.86 | 0.67 |
|  | Extramedullary disease | 3.46 | 1.63-7.39 | 0.001 |
|  | High risk cytogenetics | 1.37 | 0.66-2.85 | 0.40 |
| Overall Survival | CHIP vs. No CHIP | 0.70 | 0.21-2.28 | 0.55 |
|  | Extramedullary disease | 3.56 | 1.18-10.72 | 0.02 |
|  | High-risk cytogenetics | 1.34 | 0.45-3.92 | 0.60 |
